# Supplementary material for: Costs and cost-effectiveness of the Safer Births Bundle of Care intervention in Tanzania health facilities
Source: PLoS One. 2026 Apr 28;21(4):e0323234. doi: 10.1371/journal.pone.0323234 (PMC13123973; doi:10.1371/journal.pone.0323234)
Supplement: Attachment — Submitted filename: Response to Reviewers.docx [file pone.0323234.s001.docx]

**A RESPONSE LETTER TO ACADEMIC EDITOR AND REVIEWERS’ RESPONSE**

**Dear editor,**

Kindly receive responses raised by both academic and reviewers to the manuscript numbered **‘PONE-D-25-18416**’ entitled "Costs and Cost-Effectiveness of the Safer Births Bundle of Care Intervention in Tanzania Health Facilities."

First and foremost, we thank academic editor for thorough scrutiny of the manuscript to ensure it comply with the journal submission guidelines and standards.

Secondly, we want to thank the reviewers for revising the manuscript and provide their thoughtful comments and constructive critique that would help to improve the quality and clarity of our manuscript. We consider the revised submitted version improved.

**Academic Editor reviews and responses:**

Journal requirements:

1. Please ensure that your manuscript meets PLOS ONE's style requirements, including those for file naming. The PLOS ONE style templates can be found at

https://journals.plos.org/plosone/s/file?id=wjVg/PLOSOne_formatting_sample_main_body.pdf and

https://journals.plos.org/plosone/s/file?id=ba62/PLOSOne_formatting_sample_title_authors_affiliations.pdf

- *PLOS ONE’s style requirements have been observed and adhered.*

2. Please upload a copy of the original ethics approval letter issued by the IRB for your study. If the original letter is not in English, please also provide an English translated version in the supporting file.

- *IRB for both National Institute for Medical Research (NIMR) and that of CUHAS have been uploaded.*

Also, please report in the Methods section the dates when data were accessed for research purposes.

- *The data were accessed between on March 2024, line ‘100’.*

3. Thank you for stating the following financial disclosure:

[The author received an unconditional grant from Laerdal Foundation for PhD studies.].

At this time, please address the following queries:

a) Please clarify the sources of funding (financial or material support) for your study. List the grants or organizations that supported your study, including funding received from your institution.

-*The author received an unconditional research grant from Laerdal Foundation. There was no any material support from the funder.*

b) State what role the funders took in the study. If the funders had no role in your study, please state: “The funders had no role in study design, data collection and analysis, decision to publish, or preparation of the manuscript.”

- *The funder had no role in study design, data collection and analysis, decision to publish, or preparation of the manuscript.*

c) If any authors received a salary from any of your funders, please state which authors and which funders.

-*Corrina Vossius received a salary from the SBBC program, that was funded by the Global Facility Financing (GFF). Other authors received no specific funding for this work.*

- *No any authors received a salary from any of my funders.*

d) If you did not receive any funding for this study, please state: “The authors received no specific funding for this work.”

-*Other authors received no specific grant/salaries for this work.*

*-Corrina Vossius received a salary from the SBBC program, that was funded by the Global Facility Financing (GFF). Other authors received no specific funding for this work.*

Please include your amended statements within your cover letter; we will change the online submission form on your behalf.

4. For studies involving third-party data, we encourage authors to share any data specific to their analyses that they can legally distribute. PLOS recognizes, however, that authors may be using third-party data they do not have the rights to share. When third-party data cannot be publicly shared, authors must provide all information necessary for interested researchers to apply to gain access to the data. (https://journals.plos.org/plosone/s/data-availability#loc-acceptable-data-access-restrictions)

For any third-party data that the authors cannot legally distribute, they should include the following information in their Data Availability Statement upon submission:

1) A description of the data set and the third-party source

*- The cost data were collected from Haydom Lutheran Hospital accounting office as described in Manuscript between line ‘100 -104’. Outcome data for maternal and newborn born are described in the article of Benjamin et al., Outcomes of a Program to Reduce Birth-Related Mortality in Tanzania, DOI: 10.1056/NEJMoa2406295.*

2) If applicable, verification of permission to use the data set

-*The data can be requested from Haydom Lutheran Hospital, P.O.BOX 9000 Haydom, Manyara -Tanzania.*

*Tel. +255(0)27 253 3194/5, Fax +255(0)27 253 3734, E-mail post@haydom.co.tz*

3) Confirmation of whether the authors received any special privileges in accessing the data that other researchers would not have

-*There were no special privileges the authors received.*

4) All necessary contact information others would need to apply to gain access to the data

- *The contact information for gaining access to the data is stated under 4.5 Data sharing statement.*

5. PLOS requires an ORCID iD for the corresponding author in Editorial Manager on papers submitted after December 6th, 2016. Please ensure that you have an ORCID iD and that it is validated in Editorial Manager. To do this, go to ‘Update my Information’ (in the upper left-hand corner of the main menu), and click on the Fetch/Validate link next to the ORCID field. This will take you to the ORCID site and allow you to create a new iD or authenticate a pre-existing iD in Editorial Manager.

*-The pre-existing an ORCID iD have been authenticated in Editorial Manager.*

6. Please amend either the abstract on the online submission form (via Edit Submission) or the abstract in the manuscript so that they are identical.

-*Abstract on the online submission and that in the manuscript have been amended and they are identical.*

7. Please include a separate caption for each figure in your manuscript.

-*Separate captions for each figure have been included in the submitted manuscript.*

**Response to Reviewers**

These requirements are now been fulfilled.

**Comments from the Editor and Reviewer #2:**

Thank you very much for your positive evaluation.

1. Limited Consideration of Morbidity: DALYs were estimated but disability weights were not applied, as morbidity data were not available. An argument of potential underestimation of averted DALYs would be more transparent.

*Answer. We have now rephrased the discussion of the limitations in calculating DALYs averted. We reckon that morbidity might have been estimated both too low, (as asphyxia might cause brain damage that we have not accounted for), and too high (as the intervention might have prevented cases of asphyxia). Therefore, we might have both over- and underestimated the number of DALYs averted by not taking YLDs into account. (Discussion, lines ‘280-289’)*

2. Attribution of Outcomes: Even while the effectiveness is attributed to the SBBC intervention, clarification regarding potential confounders or co-occurring health interventions occurring over the period of study would add to causal inference.

*Answer: We have now clarified in the description of the SBBC intervention, that the SBBC implementation was the only relevant change to the health care system and treatment recommendation at this time (Introduction, page 2,’85-86’)*

3. Generalizability: Even if being used across varying levels of facilities and geographic areas, the manuscript could better clarify how findings would extend outside of the Tanzanian context, specifically to other LMICs that have varying capabilities of the health system.

*Answer: We have now added a paragraph about generalizability under Policy implication, where we state as well that the bundle is presently implemented in Nigeria (Discussion, Policy implication, page 14,’307-313’).*

4. Future Implications: The conclusion would be stronger if more focus were given to detailing anticipated Phase II and III expectations, especially financing mechanisms and health system integration.

*Answer: We have now added the suggested details about the present Phase II and planned Phase III in the Conclusion. (Conclusion, ‘318-326’)*

**Comments from Reviewer #1:**

1. 99-101 ‘Cost data were based on secondary data collected retrospectively in March 2024. Data sources were the itemized budget of the project activities and the actual spending on the itemized activities as registered in the financial records’

– Would need proof that the itemized budget was not over-estimated in the first place. This is due to the varied pricing of goods/services in different countries e.g. the price of a pencil, for example, in the US differs greatly from its price in Tanzania. Also, was the intervention standard in all cases or were there some cases where less was done? This can affect the cost perceived as well. This is not clear.

*Answer: Thank you for pointing out this important topic. The budget was compiled by the Tanzanian primary investigator and his team. It was originally expressed in Tz shilling, and only for the purpose of this paper converted to USD. To emphasize this, we stated that “All costs were estimated in Tanzanian Shillings (TZS) and converted to 2020 USD using a mean exchange rate of TZS 2,305/USD provided by the Central Bank of Tanzania” in the section Methods – Cost data collection (page 3, ‘111-113’) Insight into the budget and the actual spendings can be requested at the Haydom Lutheran Hospital, as stated in the Data sharing statement.*

119,120 ‘From the mothers, data were collected on maternal death, age, parity and whether she was referred from another hospital’

– What is the significance of the last variable? It is not mentioned anywhere else in the study.

*Answer: The variable about whether or not the mother was referred from another hospital was used in the statistical analysis of the death incidences, as data was adjusted for clinically relevant demographic and clinical factors. This is described in detail in Kamala and Ersdal at al. Outcomes of a Program to Reduce Birth-Related Mortality in Tanzania. New England Journal of Medicine. 2025.*

169,170 ‘The total estimated number of lives saved during SBBC Phase I was 580 (95% CI 225, 935) perinatal lives 382 (95% CI 138, 626) maternal lives, and 962 combined lives saved’

– Please correct the punctuation in this sentence.

– There is no mention of the baseline that existed before the intervention. This raises the question whether the trend was worse before the intervention or not.

– Were other confounding factors taken into consideration such as non-partum related deaths such as stage 4 cancer or COVID-19 in the mother? These may have increased the number of maternal deaths despite the causes not being related to delivery.

Answer: *Thank you for pointing to that important question. We have not considered non-partum related deaths. However, as this study observes mortality during birth, this will probably not be affected by severe diseases like cancer or other non-communicable diseases. As the study period lasted from 2021 to 2023, concurrent with the COVID pandemic, this is unlikely to impact the study outcome. As to best of our knowledge, there were no other epidemics in the observed regions during that time.*

269, 271 ‘In addition, this study is merely a partial economic evaluation as it only deals with the program’s perspective, not capturing all costs arising to the health care provider like staff time and utilities and leaving behind the societal part of economic evaluation.’

– Although mentioned in your study limitations, it would have been important, especially to inform policymakers, of the overall cost the intervention carries as the cost-benefits implied might be overshadowed by the overall economic cost.

*Answer: We absolutely agree with the Reviewer. The study group has just received a grant to explore the full scope of health economic consequences of the program and its spill over effects. Unfortunately, the present paper had to rely on the available information.*

With this clarification and response, we hope that we have addressed both academic editor and reviewers’ comments. We are also flexible to continue receiving any comments that would further perfect our manuscript,

Regards,

Boniphace Richard Marwa,

**Corresponding Author**
