# Supplementary material for: Costs and cost-effectiveness of the Safer Births Bundle of Care intervention in Tanzania health facilities
Source: PLoS One. 2026 Apr 28;21(4):e0323234. doi: 10.1371/journal.pone.0323234 (PMC13123973; doi:10.1371/journal.pone.0323234)
Supplement: Attachment — Submitted filename: Plos One Review Nov 27.docx [file pone.0323234.s002.docx]

1. Recommendation: Accept Minor Revision

2. Is the manuscript technically sound, and do the data support the conclusions? Yes

3. Has the statistical analysis been performed appropriately and rigorously? It appears to be

4. Does the manuscript adhere to the PLOS Data Policy? Additional details can be found at <http://journals.plos.org/plosone/s/materials-and-software-sharing>. Yes

5. Is the manuscript presented in an intelligible fashion and written in standard English? It is written in standard English

6. Review Comments to the Author (minimum 200 characters)

The article “Costs and Cost-Effectiveness of the Safer Births Bundle of Care Intervention in Tanzania Health Facilities” presents an important analysis of a phase 1 implementation of the Safer Births Bundle of Care in 30 health facilities in Tanzania from 2021 to 2023. This article presents data that was collected retrospectively. While the article mentions some information about the intervention in lines 71-78 it is difficult to understand what exactly is involved in this bundle of care. While this is an economic analysis it would be helpful to have more detail about what was involved in the intervention to give this more context.

It would also be of interest to understand how many people were involved in the training and implementation of this across these centers. Interestingly this study was done in both rural and non-rural areas with different levels of deliveries. It would be interesting if they could present some of the information on cost and cost-effectiveness in these different types of settings.

They discuss travel cost being limited due to the pandemic restrictions. It would be helpful to understand a bit more about whether the intervention facilitated travel within the region and also affected women to get referred from primary to secondary to tertiary centers if required. It would be helpful to understand the regional context a bit. Could emergency C-sections be performed in the primary center and did they have newborn resuscitation facilities in these primary centers? It would be interesting to understand how this intervention worked and the costs and cost-effectiveness in the primary vs. secondary vs. tertiary setting.

This is a commendable study and should be accepted with minor corrections.

7. PLOS authors have the option to publish the peer review history of their article (<https://journals.plos.org/plosone/s/editorial-and-peer-review-process#loc-peer-review-history>). If published, this will include your full peer review and any attached files. Do you want your identity to be public for this peer review? No
7a. If you answered "Yes" above, that you would like your identity to be public, please provide your full name, as it should appear on the published peer review. Please do not sign the review on behalf of another person.

Confidential to Editor:
1. Do you have any potentially competing interests? None

2. Did you receive any assistance in preparing this review No

3. Do you want to receive recognition for this review on a Web of Science researcher profile? No

(<https://clarivate.com/products/scientific-and-academic-research/research-discovery-and-workflow-solutions/researcher-profiles/>)? If you opt in, your Web of Science profile will automatically be updated to show a verified record of this review in full compliance with the journal's review policy. If you don't have a Web of Science profile, you will need to first create a free account. (Answer options: Yes, No)
