# Supplementary material for: Costs and cost-effectiveness of the Safer Births Bundle of Care intervention in Tanzania health facilities
Source: PLoS One. 2026 Apr 28;21(4):e0323234. doi: 10.1371/journal.pone.0323234 (PMC13123973; doi:10.1371/journal.pone.0323234)
Supplement: Attachment — Submitted filename: Response_to_Reviewers_auresp_2.docx [file pone.0323234.s003.docx]

**RESPONSE LETTER TO the ACADEMIC EDITOR AND REVIEWERS’ RESPONSE**

Dear Editor,

Kindly receive the responses to the comments of Reviewer #3 to the manuscript numbered **‘PONE-D-25-18416**’ entitled "Costs and Cost-Effectiveness of the Safer Births Bundle of Care Intervention in Tanzania Health Facilities."

First and foremost, we thank the editor for taking over the publication process and handling the manuscript in a timely manner.

Secondly, we want to thank the reviewers for revising the manuscript and provide their thoughtful comments and constructive critique that would help to improve the quality and clarity of our manuscript.

We are very happy that Reviewer #1 has no further comments.

**Comments from Reviewer #3:**

Thank you very much for your positive evaluation.

1. While the article mentions some information about the intervention in lines 71-78 it is difficult to understand what exactly is involved in this bundle of care. While this is an economic analysis it would be helpful to have more detail about what was involved in the intervention to give this more context. It would also be of interest to understand how many people were involved in the training and implementation of this across these centers.

*Answer: We have now given more detail about the main clinical targets of the intervention and the organization of the initial training as cascade training (Introduction, The Safer Births Bundle of Care, page 2, line 79 – 85.)*

2. Interestingly this study was done in both rural and non-rural areas with different levels of deliveries. It would be interesting if they could present some of the information on cost and cost-effectiveness in these different types of settings.

*Answer: This is indeed a very relevant question and similar to question 5, where you enquire about a sub-analysis of the different levels of healthcare services. Unfortunately, both topics are beyond the scope of this paper. However, we are currently planning to perform these sub-analyses.*

3. They discuss travel cost being limited due to the pandemic restrictions. It would be helpful to understand a bit more about whether the intervention facilitated travel within the region and also affected women to get referred from primary to secondary to tertiary centers if required. It would be helpful to understand the regional context a bit.

*Answer: We have now clarified that the travel restrictions due to the Corona-pandemic concerned the national and international faculty that was conducting and supervising the training. The travels of the mothers to the hospitals or during referrals was not part of the SBBC budget, and we have no data about it. However, regional travels in Tanzania were not restricted during the pandemic. (Results, Costs of the SBBC intervention, page 7, line 194)*

4. Could emergency C-sections be performed in the primary center and did they have newborn resuscitation facilities in these primary centers?

*Answer: We have now added the information that all of the health facilities provide Comprehensive Emergency Obstetric and Basic Newborn Care services and have separate labour units and operating theatres. Before the start of the intervention, we assessed the readiness of the health facilities and refurbished them, where necessary. (Introduction, The Safer Births Bundle of Care, page 2, line 90-92)*

5. It would be interesting to understand how this intervention worked and the costs and cost-effectiveness in the primary vs. secondary vs. tertiary setting.

*Answer: Please see our comment to question 2.*

Regards,

Boniphace Richard Marwa,

**Corresponding Author**
